# The Effect of Physiotherapy Intervention on an Infant With Congenital Heart Defect Associated With Developmental Delay: A Case Report

**DOI:** 10.7759/cureus.60215

**Published:** 2024-05-13

**Authors:** Chaitali S Vikhe, H V Sharath, Neha A Brahmane, Swapnil U Ramteke

**Affiliations:** 1 Department of Sports Physiotherapy, Ravi Nair Physiotherapy College, Datta Meghe Institute of Higher Education and Research, Wardha, IND; 2 Department of Pediatric Physiotherapy, Ravi Nair Physiotherapy College, Datta Meghe Institute of Higher Education and Research, Wardha, IND

**Keywords:** physical therapy, rehabilitation, physiotherapy, developmental delay, congenital heart defects

## Abstract

Congenital heart defects (CHDs) are one of the most prevalent anomalies present at birth globally. Children with CHD often face developmental challenges, including motor, language, and cognitive impairments. This case report presents the clinical profile of a 1.2-year-old female child with CHD and developmental delay (DD) post-CHD surgery. The child exhibited delayed gross motor, fine motor, language, and personal-social milestones, along with significant cardiac anomalies observed on CT angiograms. Physiotherapy interventions were initiated to address these DDs, encompassing manual techniques, neurodevelopmental treatment, and multimodal stimulation. The objective of this study was to assess the impact of physiotherapy interventions on improving developmental outcomes in infants with CHD-associated DD. The New Ballard Score and Hammersmith Infant Neurological Examination were utilized to evaluate improvements post-intervention. Significant enhancements in developmental outcomes were observed. This case underscores the significance of holistic care approaches in mitigating the impact of CHD on developmental trajectories and improving the quality of life for affected children.

## Introduction

Congenital heart defects (CHDs) represent one of the most prevalent forms of congenital anomalies, impacting around 0.8% to 1.2% of newborns globally [1]. These anomalies involve a range of structural abnormalities affecting the heart and major blood vessels, from simple anomalies to complex malformations. Around 25% of CHDs are classified as critical, necessitating surgical intervention often required within one year of life to address critical CHDs; percutaneous transcatheter intervention has become a cornerstone of therapy for CHD lesions affecting both the systemic and pulmonary vascular systems [2,3]. Although mortality rates among children with CHD have notably decreased, there is a rising cohort of individuals surviving into adulthood, highlighting the necessity to enhance long-term development and overall quality of life [4].

Developmental impairments, including intellectual difficulties, poor motor skills, attention, and speech or language disorders, are prevalent in up to 50% of children diagnosed with CHD [5,6]. Furthermore, a significant portion of these children may necessitate treatment or surgical intervention [7]. There is a notable gap in the comprehensive synthesis of motor impairments in these children, underscoring the necessity of a deeper understanding of these impairments across childhood. This need emphasizes the importance of developing targeted guidelines for the surveillance of developmental milestones in this population [8-10].

The long-term implications of CHD on neurodevelopmental outcomes are increasingly recognized as an area of concern [11]. They are especially vulnerable to experiencing developmental delays (DD) [12], which present as delays in reaching developmental milestones across various domains, including motor skills, cognition, language, and socioemotional capabilities [13]. Recent research indicates that children with CHD have a high risk of experiencing DDs compared to their healthy counterparts [14]. The underlying pathophysiological mechanisms linking CHD to neurodevelopmental impairment are complex and multifaceted [15]. These mechanisms may include factors such as prenatal hypoxia, altered cerebral blood flow, genetic predispositions, and the effects of cardiac surgery and associated treatments on the developing brain. Understanding this interplay is crucial for optimizing long-term outcomes and guiding early intervention strategies. This case report aims to contribute to the existing literature by presenting a detailed clinical profile and developmental trajectory of a 1.2-year-old child with CHD and DD.

## Case presentation

This is a case of a 1.2-year-old female child who was apparently healthy, after two months of birth, the baby appeared to be doing well. However, one night, at 10 pm, the child suddenly began crying, and her parents noticed discoloration in her lips and tongue. They took her to a private hospital at 1 am. Upon admission, she was immediately transferred to the neonatal intensive care unit (NICU) and was on a ventilator for four days after being diagnosed with patent ductus arteriosus (PDA). Two days after being diagnosed with PDA, her parents brought her to Acharya Vinoba Bhave Rural Hospital (AVBRH), Wardha, India, and on November 29, 2022, she underwent arterial switch surgery; for eight days, she was on a ventilator with concerns about her low birth weight. On postoperative day 3, she developed the infection which was later resolved with proper treatment (including the removal of excess fluid). She eventually recovered and was discharged from the hospital. Eight months later, at the age of one year, on September 26, 2023, her parents returned to the hospital with concerns about her inability to lift her neck, tilt, or sit properly with and without support. Following investigations, including CT scans, and neurological assessments and then she was diagnosed with delayed developmental milestones. Her parents then brought her for physiotherapy treatments at the pediatric physiotherapy OPD. Upon assessment, her developmental age was three months, whereas her chronological age was 1.2 years.

Clinical findings

Before the examination, the patient's informed consent was taken, after which a physical examination was conducted. During the examination, the highest attainable functional position was sitting without support. However, developmental milestones were not achieved according to her developmental age. A detailed timeline of events is provided in Table 1.

**Table 1:** Timeline of events AVBRH: Acharya Vinoba Bhave Rural Hospital

Events	Timeline
Date of birth of the child	September 14, 2022
Visited AVBRH for the above-mentioned complaints	November 27, 2022
Underwent arterial switch surgery	November 29, 2022
Again visited AVBRH for a follow-up	September 26, 2023
Physiotherapy assessment is taken of the child	November 1, 2023

Developmental milestones related to gross motor skills were not attained. A detailed overview of gross motor development is provided in Table 2.

**Table 2:** Developmental milestones related to gross motor skills

Gross motor	Normal	Attained month
Head control	6 weeks	Partially attained
Rolling	4-6 months	Not attained
Sitting	5-7 months	Not attained
Creeping	6-8 months	Not attained
Crawling	9-11 months	Not attained
Standing with support	9-12 months	Not attained

Table 3 shows fine motor skills. The grasp reflex was attained at 11 months, but reaching, releasing, mouthing, transferring, and grasping were not achieved within the expected timeframes.

**Table 3:** Developmental milestones associated with fine motor skills

Fine motor	Normal	Attained month
Grasp reflex	0-3 months	11 months
Reach	2-4 months	Not attained
Release	3-6 months	Not attained
Mouthing	3-6 months	Not attained
Transfer	4-5 months	Not attained
Grasp	6-8 months	Not attained

Table 4 shows language acquisition. Turning head to sound was attained at six weeks, and cooing at three months, but monosyllables and disyllables were not achieved at six and nine months, respectively.

**Table 4:** Developmental milestones related to language acquisition

Language	Normal	Attained month
Turns head to sound	6 weeks	40 weeks
Cooing	3 months	12 months
Monosyllables	6 months	Not attained
Disyllables	9 months	Not attained

Table 5 shows personal and social interactions. Social smile was attained at one month, recognizing mother at three months, but smiling at mirror image and waving bye-bye were not achieved at six and nine months, respectively.

**Table 5:** Developmental milestones associated with personal and social interactions

Personal and social	Normal	Attained month
Social smile	1 month	9 months
Recognizing mother	3 months	11 months
Smiles at the mirror image	6 months	Not attained
Waves Bye-Bye	9 months	Not attained

Table 6 shows primitive reflexes. The sucking reflex was present immediately after birth, the Moro reflex was present at 4-6 months, but the crossed extension was absent at two months, and other reflexes were integrated within the expected timeframes.

**Table 6:** Various reflexes observed in infants and their typical developmental timeline ATNR: asymmetrical tonic neck reflex

Reflexes	Normal	Present/integrated
Sucking reflexes	Immediately after birth	Present
Moro reflexes	4-6 months	Present
Grasp reflexes	Immediately after birth	Present
Flexor withdrawal	2 months	Present
Extensor thrust	2 months	Present
Crossed extension	2 months	Absent
Startle	3 months	Present
ATNR	6 months	Present

Investigations

**Figure 1:**
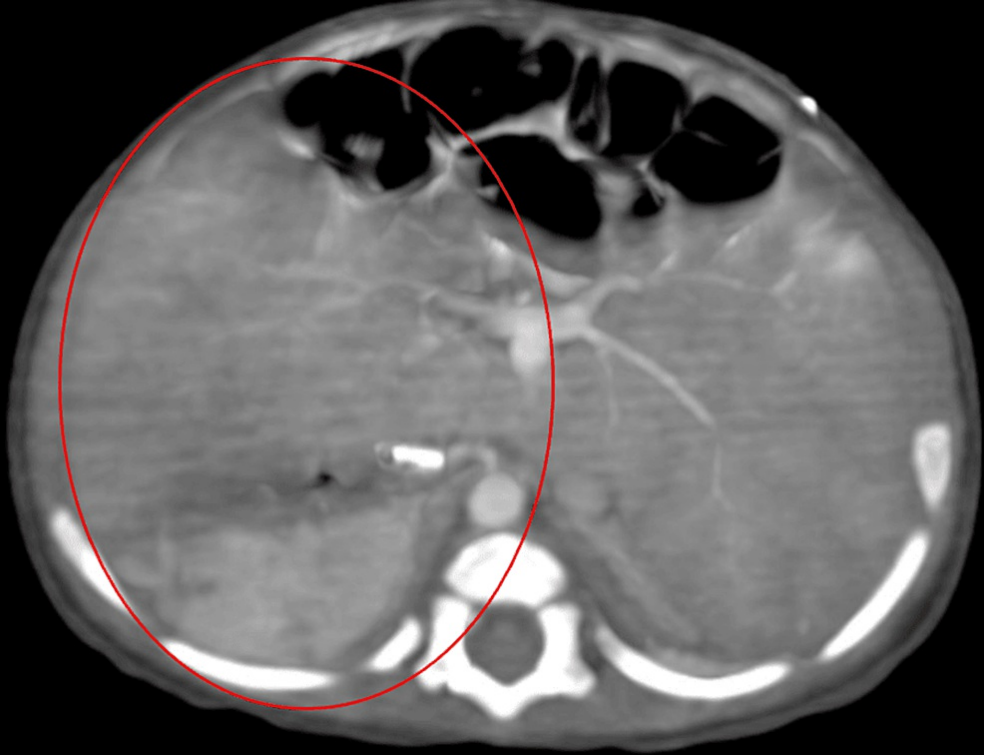
CT angiogram of the pulmonary arteries

CT angiogram of the pulmonary arteries shows significance as it indicates an abnormal positioning of major blood vessels within the cardiovascular system. The right-sided aorta suggests a reversal of the usual orientation, which can impact blood flow dynamics and cardiac function as shown in Figure 1.

Physiotherapy intervention

**Figure 2:**
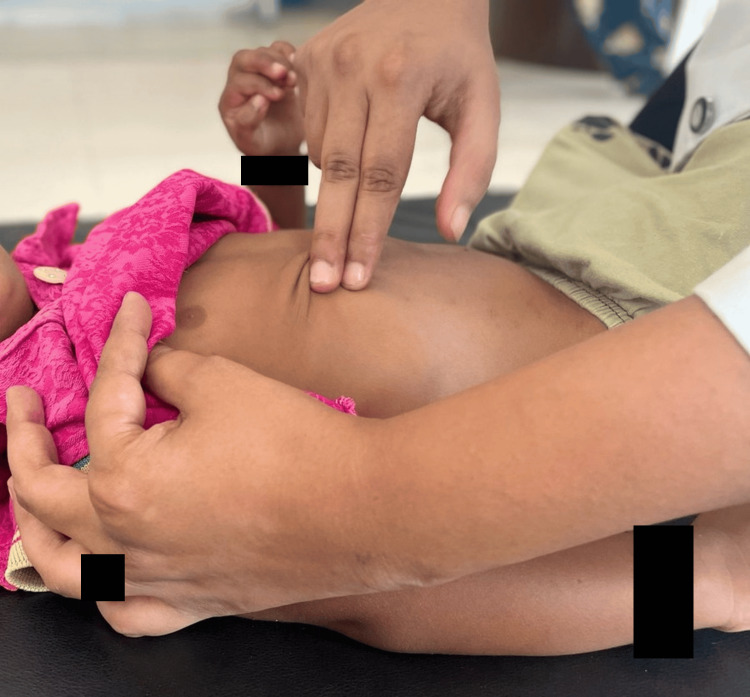
Manual chest vibration for different lobes

In Table 7, we mentioned a tailored rehabilitation protocol that included respiratory and neuromuscular function-related strategies. Rehabilitation went on for six months daily for an hour and strategies were reached to the primary caregiver that should be done at home [15]. Manual chest vibration is shown in Figure 2.

**Table 7:** Physiotherapy intervention

Goals	Intervention	Techniques	Procedure	Intensity	Dosage
To airway clearance	Manual chest percussion	Apical, middle, basal	Percussion should be given on your lap to children. Apical - The patient should be in a sitting or semi-reclined position with their back supported at a 30-degree angle. Percussion is performed between the clavicle and the top of the scapula on each side. Middle - The patient should be in a supine position with the lower end of the body elevated to about 12 inches. Percussion over the mid-lung fields. Basal - The patient should be in a prone position, and percussion is performed over the lower lung fields.	Moderate	5 minutes on each side
To facilitate secretion clearance and to Improve respiratory function	Manual chest vibration (Figure 2)	Apical, middle, basal	This should be applied to the chest wall, using the fingertips.	Low to moderate	5 minutes on either side
To enhance postural control and to improve sensory integration and proprioception	Neurodevelopmental technique	Neck-holding, rolling, weight-bearing	Neck holding - On the plain couch in the prone position. Progression - On-side lying position On wedge with auditory and visual stimulation. On vestibular ball in supine, prone.	Low to moderate	10 minutes
			Rolling - On either side of the plain couch On a wedge with auditory and visual stimulation.	Low to moderate	10 minutes
			Weight-bearing - Prone on elbows with auditory and visual stimulation.	Low to moderate	10 minutes
To increase sensory awareness	Multimodal stimulation	Tactile stimulation, gentle rocking	Tactile stimulation - gentle stroking over the face, upper limb, lower limb, back, and trunk. Gentle rocking- sideways, backward and forward.	Low	10 minutes

Outcome measures

The study incorporated various outcome measures to evaluate gross motor functions, neurological examination, and musculoskeletal system both before (pre) and after (post) six months of physiotherapeutic interventions. Table 8 outlines the pre- and post-physiotherapy rehabilitation outcomes, indicating significant improvements across the assessed parameters.

**Table 8:** Outcome measures before intervention and after intervention The New Ballard Score is an assessment utilized to estimate the gestational age of newborns particularly preterm infants, based on physical and neurological characteristics, It evaluates various physical and neuromuscular maturity indicators such as skin texture, lanugo, posture, reflexes, and genital development, This tool aids in clinical decision-making and planning for neonatal care [16]. The Hammersmith Infants Neurological Examination is used to evaluate the neurological function and development of infants, particularly those with neuromuscular disorders. It assesses motor function, muscle tone, reflexes, and other neurological signs, providing valuable insights into developmental milestones and potential neurological impairments early in life [17].

Sr no.	Outcome measure	Pre-treatment	Post-treatment
1.	New Ballard Score	17	35
2.	Hammersmith Infants Neurological Examination	25	44

## Discussion

The case report delving into the effect of physiotherapy intervention on infants with CHDs associated with DDs presents a compelling narrative of holistic care. Infants born with CHDs often face developmental challenges due to compromised oxygen supply and cardiac function. This case underscores the pivotal role of physiotherapy in addressing both the cardiac and developmental aspects of these infants' health. By integrating targeted exercises, developmental stimulation, and close monitoring, the physiotherapy intervention not only aims at improving cardiac function but also fosters optimal growth and developmental milestones. Furthermore, the report highlights the multidisciplinary approach essential to managing complex medical conditions in infants. Collaborative efforts between cardiologists, pediatricians, and physiotherapists ensure comprehensive care tailored to the specific needs of each patient. The integration of physiotherapy within this framework signifies its significance beyond traditional rehabilitation, extending to the realm of early intervention and developmental support. Through close coordination and communication among healthcare professionals, this approach maximizes the potential for positive outcomes and enhances the quality of life for infants with CHDs and DDs [18].

The presented case scenario is of a 1.2-year-old female child with congenital heart disease, which represents a complex spectrum of structural anomalies affecting the heart and major blood vessels. The relationship between CHD and DD is multifaceted and multifactorial. The positive points presented in the assessment showed a delay in developmental milestones, including gross motor, fine motor, language, and social milestones. Persisted primitive reflexes and constant secretions in all three lobes of bilateral lungs were assessed on auscultation [16]. The suggestive CT angiogram of the pulmonary artery findings showed abnormal positioning of major blood vessels within the cardiovascular system and a right-sided aorta, suggesting a reversal of the usual orientation. And also hypertrophy of the muscles of the left ventricles with the atrophied left atrium, which leads to an increased workload on the heart. Therefore, there was a need for pediatric physiotherapy protocols to enhance and improve the developmental milestones; neurodevelopmental techniques were given. Airway clearance techniques included manual chest percussion, manual chest vibrations, and multimodal stimulation, which included tactile and vestibular systems [19,20].

Physiotherapy interventions play a crucial role in addressing the developmental challenges faced by children with CHD. These interventions are tailored to target specific motor deficits and promote optimal developmental trajectories. Manual techniques, such as chest percussion and vibration, aim to improve respiratory function and facilitate secretion clearance. It is unclear how precisely percussion could aid in the evacuation of secretions. Intrathoracic pressure rises with mechanical percussion. However, no research has been done to look at the impacts of physical percussion. According to a theory, secretions stuck to the walls of the airways are released when a vibratory wave is produced by the air trapped between the cupped hand and the chest wall [21].

Neurodevelopmental treatment focuses on enhancing motor learning and skill acquisition through structured exercises. These interventions aim to improve muscle strength, coordination, and motor control, addressing gross motor delays commonly observed in children. Additionally, multimodal stimulation techniques provide sensory input to promote sensory processing and motor integration, fostering a conducive environment for developmental progress. It's essential to recognize the importance of early intervention in mitigating the impact of DDs associated with CHD [22,23]. Early identification of delays and prompt initiation of physiotherapy interventions can significantly improve outcomes and enhance the overall quality of life for these children. Moreover, ongoing assessment and adjustment of intervention strategies are crucial to address evolving developmental needs and optimize long-term outcomes.

Moreover, the case report sheds light on the importance of individualized care plans in addressing the unique challenges faced by each infant with CHD and DDs. Physiotherapy interventions are tailored to the infant's cardiac condition, developmental stage, and response to treatment, emphasizing the personalized nature of care. By continuously assessing progress, adjusting interventions, and providing family-centered support, physiotherapists play a crucial role in optimizing outcomes and empowering families to actively participate in their child's care journey. This case report serves as a testament to the transformative impact of physiotherapy on promoting holistic well-being and fostering resilience in infants facing complex medical conditions from the earliest stages of life. While the case report demonstrates the positive impact of physiotherapy on infants with CHDs and DDs, its findings are limited by the lack of a control group, reliance on a single case, and a short follow-up period, which further constrain the study. However, it highlights the importance of interdisciplinary collaboration and early intervention in optimizing outcomes for infants with CHD. Future research with larger samples and longitudinal designs is needed to validate these findings and guide evidence-based interventions for this vulnerable population.

## Conclusions

This case report highlights the critical role of physiotherapy in addressing the DDs associated with CHD. Through targeted interventions and objective outcome measures, significant improvements in developmental outcomes can be achieved. By advocating for holistic care approaches that integrate cardiac management with early intervention strategies, this study contributes to the advancement of care practices for children with CHD, ultimately aiming to optimize their long-term well-being and quality of life.

## References

[1] Wu W, He J, Shao X (2020). Incidence and mortality trend of congenital heart disease at the global, regional, and national level, 1990-2017. Medicine (Baltimore).

[2] Sprong MC, Broeders W, van der Net J, Breur JM, de Vries LS, Slieker MG, van Brussel M (2021). Motor developmental delay after cardiac surgery in children with a critical congenital heart defect: a systematic literature review and meta-analysis. Pediatr Phys Ther.

[3] Srushti Sudhir C, Sharath HV (2023). A brief overview of recent pediatric physical therapy practices and their importance. Cureus.

[4] Jones CE, Desai H, Fogel JL (2021). Disruptions in the development of feeding for infants with congenital heart disease. Cardiol Young.

[5] Bonthrone AF, Chew A, Kelly CJ (2021). Cognitive function in toddlers with congenital heart disease: the impact of a stimulating home environment. Infancy.

[6] Huisenga D, La Bastide-Van Gemert S, Van Bergen A, Sweeney J, Hadders-Algra M (2021). Developmental outcomes after early surgery for complex congenital heart disease: a systematic review and meta-analysis. Dev Med Child Neurol.

[7] Loblein HJ, Vukmirovich PW, Donofrio MT, Sanz JH (2023). Prevalence of neurodevelopmental disorders in a clinically referred sample of children with CHD. Cardiol Young.

[8] Ankar P, Sharath HV, Chavan N (2024). A case report of pediatric rehabilitation for hypoxic ischemic encephalopathy associated with global developmental delay. Cureus.

[9] Bolduc ME, Rennick JE, Gagnon I, Majnemer A, Brossard-Racine M (2022). Canadian developmental follow-up practices in children with congenital heart defects: a national environmental scan. CJC Pediatr Congenit Heart Dis.

[10] Uzark K, Smith C, Donohue J, Yu S, Romano JC (2017). Infant motor skills after a cardiac operation: the need for developmental monitoring and care. Ann Thorac Surg.

[11] Wernovsky G, Licht DJ (2016). Neurodevelopmental outcomes in children with congenital heart disease - what can we impact?. Pediatr Crit Care Med.

[12] Mussatto KA, Hoffmann RG, Hoffman GM, Tweddell JS, Bear L, Cao Y, Brosig C (2014). Risk and prevalence of developmental delay in young children with congenital heart disease. Pediatrics.

[13] Choo YY, Agarwal P, How CH, Yeleswarapu SP (2019). Developmental delay: identification and management at primary care level. Singapore Med J.

[14] Mithyantha R, Kneen R, McCann E, Gladstone M (2017). Current evidence-based recommendations on investigating children with global developmental delay. Arch Dis Child.

[15] Liamlahi R, Latal B (2019). Neurodevelopmental outcome of children with congenital heart disease. Handb Clin Neurol.

[16] Morton PD, Ishibashi N, Jonas RA (2017). Neurodevelopmental abnormalities and congenital heart disease: insights into altered brain maturation. Circ Res.

[17] Lee FT, Seed M, Sun L, Marini D (2021). Fetal brain issues in congenital heart disease. Transl Pediatr.

[18] Marino BS, Lipkin PH, Newburger JW (2012). Neurodevelopmental outcomes in children with congenital heart disease: evaluation and management: a scientific statement from the American Heart Association. Circulation.

[19] Christian PS (2014). Chest physiotherapy for infants. Int J Physiother Res.

[20] Shakya S, Parsekar SS, Ramachandran S (2022). Physiotherapy interventions for head and trunk control in children with developmental disabilities: a scoping review protocol. F1000Res.

[21] Saikia D, Mahanta B (2019). Cardiovascular and respiratory physiology in children. Indian J Anaesth.

[22] Ceran B, Beşer E, Karaçağlar NB, Beyoğlu R, Şimşek GK, Canpolat FE, Kutman HG (2021). Evaluation of the correlation of the new Ballard scoring with the ultrasonographic optical nerve sheath diameter and brain volume of preterm infants. Early Hum Dev.

[23] Romeo DM, Cowan FM, Haataja L (2021). Hammersmith infant neurological examination for infants born preterm: predicting outcomes other than cerebral palsy. Dev Med Child Neurol.

